# Dr. Samuel Buckley

**Published:** 1910-06-11

**Authors:** 


WE have to record the death of Dr. Samuel Buckley at Manchester in his 64th year. In 1868 he became a Member and in 1873 a Fellow of the R.C.S., which was followed in 1880 by his gaining the M.B. of London, of which University he graduated M.D. in 1885. He was also a B.Sc. (Hon.) of Victoria University. He was President of many scientific societies, and his appointments included the posts of hon. physician to the Manchester North Hospital for the Diseases of Women and Children, and resident medical officer in the medical and fever wards of the Manchester Royal Infirmary. He was a fairly frequent contributor to the medical papers.

